# Supplementary material for: Controlled Enzyme Cargo Loading in Engineered Bacterial Microcompartment Shells
Source: Biochemistry. 2025 Mar 5;64(6):1285–92. doi: 10.1021/acs.biochem.4c00709 (PMC11924220; doi:10.1021/acs.biochem.4c00709)
Supplement: Supplementary file 1 — bi4c00709_si_001.pdf [file bi4c00709_si_001.pdf]

Supporting Information

Controlled enzyme cargo loading in engineered bacterial microcompartment shells

Nicholas M. Tefft[1], Yali Wang[2], Alexander Jussupow[1], Michael Feig[1], and Michaela A. TerAvest[1]*

[1]Department of Biochemistry and Molecular Biology, Michigan State University, East Lansing, MI, USA, 48824

[2]Department of Microbiology, Genetics, and Immunology, Michigan State University, East Lansing, MI, USA, 48824

*Corresponding author: teraves2@msu.edu

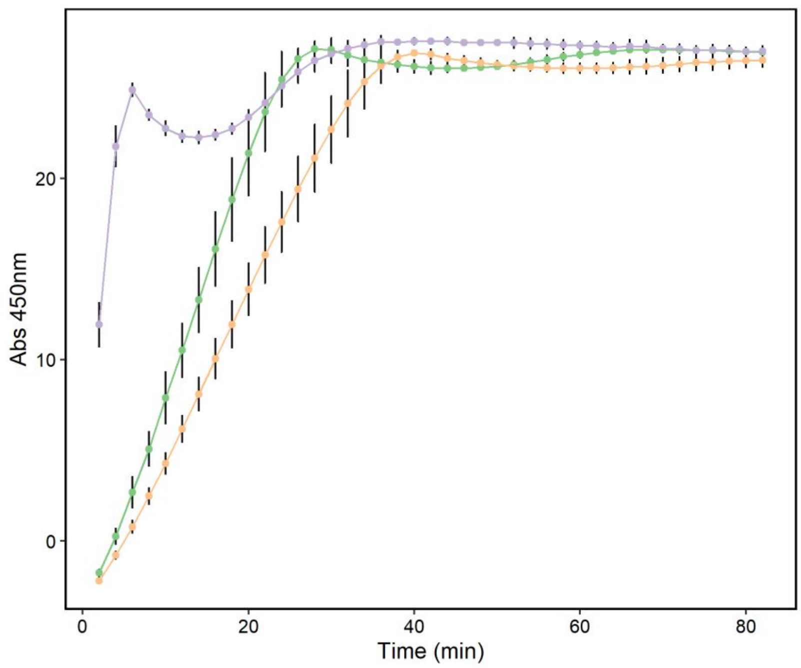

Figure S1. **TPI activity of purified SpyCatcher-TPI.** Purple: 238 ng SpyCatcher TPI. Green: TPI positive control . Orange: 23.8 ng SpyCatcher TPI.

TableS1. Dynamic Light Scattering analysis of shell samples.

Shell	Average diameter	stdev
HT1P	37.6	1.6
HT1T2T3P	39.7	1.8
HT1	49.1	1.5
HT1T2T3	43.2	0.8
